# O20 - Human rhinovirus replication-dependent induction of micro-RNAs in human bronchial epithelial cells

**DOI:** 10.1186/2045-7022-4-S1-O20

**Published:** 2014-03-14

**Authors:** Spyridon Megremis, Styliani Taka, Anastasis Oulas, Georgios Kotoulas, Ioannis Iliopoulos, Nikolaos G Papadopoulos

**Affiliations:** 1 Allergy Department, 2nd Pediatric Clinic, University of Athens, Athens, Greece; 2 Institute of Marine Biology, Biotechnology and Aquaculture - HCMR, Heraklion, Crete, Greece; 3 Division of Medical Sciences, University of Crete, Heraklion, Crete, Greece

## Background

Micro-RNAs (miRNAs) are a class of small non-coding RNA molecules that function though post transcriptional regulation of gene expression by a process termed RNA interference (RNAi). RNAi–mediated targeting of viral RNAs is recognized as an antiviral defense mechanism. The epigenetic effect of miRNAs can either be direct, by interfering with virus genome, or indirect, through downregulation of type I IFN genes. The aim of this study is to identify HRV-A1B specific miRNAs in human broncheal cell line.

## Method

*In silico* prediction of potential HRV-A1B specific human mature miRNAs was performed using two different prediction tools, miRBase and RNAhybrid. Human bronchial epithelial cells (BEAS-2B) were infected with HRV-A1B (1 MOI) along with UV inactivated HRV1B (1 MOI), zymosan (TLR4 stimulator) and Poly I:C (TLR3 stimulator). RNA was isolated at different time points and the kinetics of 8 miRNAs were evaluated. The expression of miRNAs was measured by miRNA specific RT-QPCR. The results were calculated according to the 2^-ΔΔCT method (FI). Statistical analysis was performed using Student’s t test.

## Results

Sixty two miRNAs were predicted to bind to the HRV-A1B positive strand. Eight miRNAs were selected according to their binding properties. We found replication dependent HRV-A1B specific induction in hs-miR-a (50 FI) and miR-b (24 FI) at 7 hours after HRV1B infection.

## Conclusion

To our knowledge**,** this is the first study to demostrate replication dependent induction of HRV-A1B specific human miRNAs in human broncheal epithelial cell line. The expression levels of hs-miR-a and hs-miR-b were HRV replication-dependent. Further experiments are needed in order to define the potential antiviral activity of the above miRNAs.

